# Retrospective study to identify associations between clinician training and dental implant outcome and to compare the use of MATLAB with SAS

**DOI:** 10.1186/s40729-019-0182-6

**Published:** 2019-08-09

**Authors:** Jyoti Sonkar, Pooja Maney, Qingzhao Yu, Archontia Palaiologou

**Affiliations:** 1 University of New England, College of Dental Medicine, 716 Stevens Ave, Goddard Hall 316, Portland, ME 04103 USA; 2 Department of Periodontics, Louisiana State University School of Dentistry, New Orleans, LA USA; 3 Biostatistics Program, School of Public Health, Louisiana State University Health Science Center, New Orleans, LA USA; 4 Graduate Periodontics, UT Health San Antonio, San Antonio, TX USA

**Keywords:** Dental implant outcome, Residency, Clinician training, Predictors

## Abstract

**Background:**

The aim of this study was to identify any associations between predictor variables, mainly clinician training and dental implant outcome, among the residents in different departments and to compare statistical analysis with the use of MATLAB R2017a™ to SAS version 9.4.

**Methods:**

Dental records were reviewed from January 1, 2011, to December 31, 2015. Two thousand forty-eight dental implants were placed on 471 patients seen by residents from the departments of Periodontics, Prosthodontics, and Oral and Maxillofacial Surgery (OMFS) at Louisiana State University Health Sciences Center School of Dentistry. The following parameters were investigated by means of multilevel logistic regression analysis: demographics, implant parameters, department, and residents’ year of training.

**Results:**

A total of 1449 implants were included in the study. Overall, within a 1–5-year time period, 1343 (92.6%) implants had survived and 106 (7.4%) implants failed. Discipline (*p* = 0.0004), residents’ year of training (*p* < 0.0001), and implant systems (*p* = 0.0024) showed significant associations with implant outcome. Periodontics had a survival rate of 94.14% followed by Prosthodontics (91.48%) and OMFS (89.64%). The survival rates of implants by year of training were as follows: third-year Periodontics and OMFS (94.20%), second-year (89.38%), and first-year (88.6%).

**Conclusion:**

The level and type of clinician training had an impact on implant outcome in different residency programs. Further studies will be necessary to identify the reasons for the differences in implant failure rates.

## Background

Dental implants are a predictable and commonly used option for the replacement of missing teeth. Implants are surgically placed in the alveolar processes and eventually integrate with the surrounding bone within 8–10 weeks [1]. A healthy implant is non-mobile and asymptomatic, with healthy peri-implant tissues surrounding it [1–4]. Dental implants have low failure rates of 2–5% [5]. For the purpose of this study, implant failure was defined as any condition that leads to the loss of the dental implant. Regardless of their overall high survival rate, failure of an implant is a very negative experience for the patient [5]. This may result in an increased number of surgical procedures, alveolar bone loss and further limitations in future retreatment. This is expensive for the patient as well as for the surgeon. To help put the low percentage of failures in perspective, it is important to note that based on a report by the American Academy of Implant Dentistry approximately 30 million Americans are missing teeth and one tenth of them have dental implants [6]. In 2006, about five and a half million implants were placed in the USA [6]. Even if the failure rate of these implants is only 2 percent, we can expect over 275,000 implant failures each year [6]. With an increasing number of dentists placing implants, it is evident that limiting implant failures should be a treatment goal for the modern dental practice. It is evident that implant failures pose a significant problem for both the patients and the dental practitioners [7]. There are several known systemic and local factors that may contribute to implant failure [3, 8]. For example, higher implant failure rates have been associated with poorly controlled diabetics [9]. Data suggests that smoking results in a threefold increased risk of implant failure [10]. Higher failure rates have been found in the posterior maxilla due to poor bone quality [2, 11].

The outcome of dental surgical procedures depends on various factors. One such factor is the level of experience of the surgeon. Previous studies on the outcome of periodontal disease treatment have reported the existence of a relationship between the level of training and clinical outcomes following certain periodontal procedures. Greater operator experience was associated with superior results following surgical procedures [12, 13]. However, to our knowledge, no study has explored the association of operator experience or the level of resident training with the failure rate of dental implants. Furthermore, there are no studies that have compared implant outcomes between residents trained in different specialties. If an association between the level of training and implant outcome exists, such a finding could influence the curriculum within training programs. The main objective of this study was to determine whether the level and type of clinical training had any influence on implant outcome. We hypothesized that an increased level of clinician training would significantly decrease implant failure rates. Specifically, we aimed to identify any associations between predictor variables and dental implant outcomes among the residents in the departments of Periodontics, Prosthodontics and Oral and Maxillofacial Surgery (OMFS). A secondary aim of the study was to compare the statistical analysis using two different software (SAS version 9.4 and MATLAB R2017a™) to determine if there were any differences in the results. Our hypothesis was that MATLAB Statistical Toolbox will provide more accurate results due to the fact that it allows complete assessment of the data set and provides suggestions on numerous statistical tests.

To our knowledge, MATLAB has not been routinely used for statistical analysis in the dental literature, due to its cost and the fact that it does not allow common sharing among users [14, 15].

## Methods

Retrospective analysis of records for 2048 patients who received dental implants between January 1, 2011, and December 31, 2015, at Louisiana State University Health Science Center (LSUHSC) School of Dentistry was completed. This study was approved by the LSUHSC Institutional Review Board (IRB#9397).

### Inclusion criteria

Patients between 18 and 80 years of age whose records indicated that they received dental implants between January 1, 2011, and December 31, 2015.

### Exclusion criteria

The exclusion criteria are the following:

- Patients whose implants presented with mobility at the time of placement and were removed
- Patients with incomplete records
- Patients with no follow up for at least 1 year or more after implant placement

### Determination of implant survival

Implant survival was defined as the presence of the implant at the time of evaluation of records. The data collected from the patient records included demographics (age and gender), implant site characteristics (site, graft, sinus elevation, implant system, length, and diameter), and two known confounding factors (smoking and diabetes). The study variables were the specialty, year of resident training, and implant system. Based on the inclusion and exclusion criteria, a total of 1449 implants were included in the study. Survival or failure of an implant was based on the presence or absence of the implant at 1 year after placement. The data on implant survival or failure was categorized by the specific department of the resident who placed the implant (Periodontics, OMFS, or Prosthodontics) and by the year of residency training.

### Statistical analysis

Data analysis was completed using the Statistical and Machine Learning toolbox in MATLAB R2017a™ (Mathworks Inc., Natick, MA) and SAS version 9.4 (SAS Institute Inc., Cary, NC) Implant outcome (success or failure) was selected as a dependent variable. Confounding variables included age, gender, site, smoking, diabetes, hypertension, previous graft, previous sinus lift procedure, implant system, implant length, implant diameter, immediate versus delayed placement, and past periodontal history. Study variables included the department at LSUHSC School of Dentistry and level of training. To aid in analysis, variables were expressed in binary measures, 1 corresponding to implant success and 0 corresponding to implant failure. Multiple logistic regression, comparing the implant outcome to the independent variables, was performed to establish the presence of any statistically significant associations. Multilevel logistic regression was then performed on the statistically significant associations (department, resident year of training, and implant systems). The individual clinician was used as a random variable in the model to assess any relevant finding. Clinicians were stratified into three groups based on the level of their clinical expertise. All the groups treated a similar patient population with respect to medical status, age, and gender.

Group 1 (beginners) is first-year residents in the Periodontics department and in the Prosthodontics fellowship program. At LSUHSC School of Dentistry, the Periodontics residents and Prosthodontics fellows are trained in implant placement within 6 months of their training and were exposed to a similar patient population.

Group 2 (intermediate) is second-year residents in the Periodontics department at LSUHSC School of Dentistry.

Group 3 (advanced) is third-year residents in the Periodontics and OMFS departments at LSUHSC School of Dentistry. The third year of residency was selected for both OMFS and Periodontics residents as both groups treat implant cases of similar severity at that point in their residency program.

All tests of significance were evaluated at the 5% significance level.

## Results

The statistical analysis through both MATLAB R2017a™ and SAS version 9.4 yielded significant *p* values for variables—discipline, implant system, and training by year. Survival rate was 92.6% (1343 implants) and failure rate was 7.4% (106 implants). Six hundred and seventy-two males and 777 females were included in the study with a mean age of 59.65 years. Table 1 shows the population demographics examined in this study outlining the distribution of implant outcomes. Preliminary analysis revealed a significant relationship between implant outcome and LSUHSC School of Dentistry clinical department, implant system, and year of training (Table 2). Other confounding variables such as age, gender, hypertension, diabetes, smoking, and implant characteristics, did not significantly influence the implant survival or failure rate.

**Table 1:** Population demographics outlining the distribution of implant outcomes. Mean age of the population 59.65 years (minimum 18 years; maximum 94 years)

	Success *n* (%)	Failure *n* (%)
Gender		
Male (672)	626 (93.15)	46 (6.85)
Female (777)	716 (92.14)	61 (7.86)
Hypertension		
Yes (608)	555 (91.28)	53 (8.72)
No (841)	787 (93.57)	54 (6.43)
Diabetes		
Yes (212)	190 (89.62)	22 (10.38)
No (1237)	1152 (93.12)	85 (6.88)
Smoking		
Yes (249)	228 (91.56)	21 (8.44)
No (1200)	1114 (92.83)	86 (7.17)
Graft		
Yes (613)	566 (92.33)	47 (7.67)
No (836)	776 (92.82)	60 (7.18)
Sinus		
Yes (141)	126 (89.36)	15 (10.64)
No (1308)	1216 (92.96)	92 (7.04)

*n* total number of implants placed

**Table 2:**
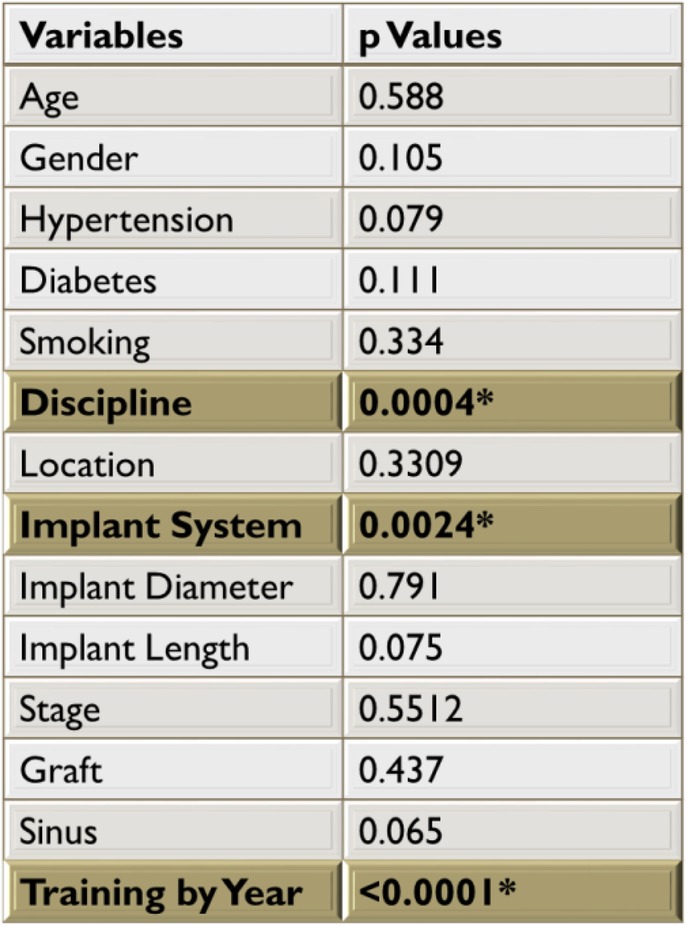
Multiple logistic regression comparing each variable to implant outcome

*Multilevel logistic regression comparing the implant outcome to discipline, implant, and year of training using each clinician as a random variable

The analysis indicated that implants were more likely to fail when placed by less experienced clinicians, when compared to more experienced clinicians (*p* = 0.0001, Tables 2 and 3). Similarly, the LSUHSC School of Dentistry Department of Training had a strong association with implant outcome (Tables 2 and 4). Residents who were trained in Periodontics had a lower implant failure rate compared with those trained in Prosthodontics and OMFS (*p* = 0.0004). Periodontics residents placed a higher number of implants (*n* = 887) and had an implant success rate of 94.14%. Prosthodontics residents placed 176 implants and had a success rate of 91.48%, and OMFS residents placed 386 implants and achieved a success rate of 89.64%.

**Table 3:** Distribution of Implant outcomes amongst clinicians through various level of training

	Survival *n* (%)	Failure *n* (%)
Group 1 (193) beginners	171 (88.6)	22 (11.4)
Group 2 (273) intermediate	244 (89.38)	29 (10.62)
Group 3 (983) advanced	926 (94.20)	57 (5.80)

*n* total number of implants placed

**Table 4:** Distribution of implant outcomes among various disciplines

	Survival *n* (%)	Failure *n* (%)
Periodontics (887)	835 (94.14)	52 (5.86)
Prosthodontics (176)	161 (91.48)	15 (8.5)
Oral and Maxillofacial Surgery (386)	346 (89.64)	40 (10.36)

*n* total number of implants placed

## Discussion

Based on the inclusion and exclusion criteria, 1449 implants were included in the study. One of the objectives of this study was to understand whether the training and resident experience along with two previously established risk factors for implant failure affected the implant outcome. Several parameters such as age, gender, systemic conditions such as hypertension and diabetes, smoking, location, native bone versus grafted bone, one-stage versus two-stage implant placement, implant characteristics (length and diameter), resident training levels, and department of training were analyzed. Smoking, diabetes mellitus, implant site characteristics, and geometry did not yield any significant association with implant failures.

The overall survival rate of the endosseous implants placed in all three departments at LSUHSC School of Dentistry was 92.6% (1343 out of 1449 implants). Our findings are lower compared with implant survival rates previously reported at other university settings [16–18]. This could be attributed to the fact that our overall survival rates were calculated based on the implant outcomes from multiple departments (Periodontics, Prosthodontics, OMFS) and levels of surgical training rather than a single department. The implant survival rates within the different departments were 94.14% for Periodontics, 89.64% for OMFS, and 91.48% for Prosthodontics. These survival rates are consistent with previously published reports [17, 19, 20].

The implant comparisons made also sought to determine whether length and diameter influenced the success rate of the implant. This study included a variety of implant lengths ranging from 6 to 16 mm and diameters ranging from 3.25 to 6 mm. The implant dimensions did not show any significant associations with implant outcome in our study. This finding is in agreement with some published data on the success of short implants [16, 21–24].

A potential contributing factor for the higher success rates found for the Department of Periodontics may be the more intensive maintenance plan followed by that department. The department of periodontics follows a very strict maintenance program for all implant patients. Evidence in the literature has shown that regular maintenance intervals of 3–4 months in the first year of implant placement and 6–12 months afterwards is critical for dental implant survival. This supports the theory that implant success is positively influenced by regular maintenance of dental implants [25–28].

This study further aimed to identify potential associations between types of clinical training and implant outcomes. Overall, the advanced group (94.2%) had the best implant outcomes followed by the intermediate group (89.38%) and beginner group (88.6%). Our results would seem to indicate that increased clinician training improves clinical outcomes; these findings are consistent with previously published periodontal treatment studies that reported a positive association between increased clinician experience and improved outcomes of periodontal procedures [12, 29–31]. The study completed by Lambert, reports that clinicians who placed more than 50 implants were 2 times more likely to have a better clinical outcome compared with inexperienced clinicians [32]. A university-based study, however, did not find a significant influence of operator experience on dental implant outcomes [33]. The study reported a 91% survival rate of implants placed by OMFS residents in their various level of training which was followed for over 6 months [33]. Similarly, another article reported an unremarkable association in implant outcomes between novice and experienced operators [34].

**Fig. 1:**
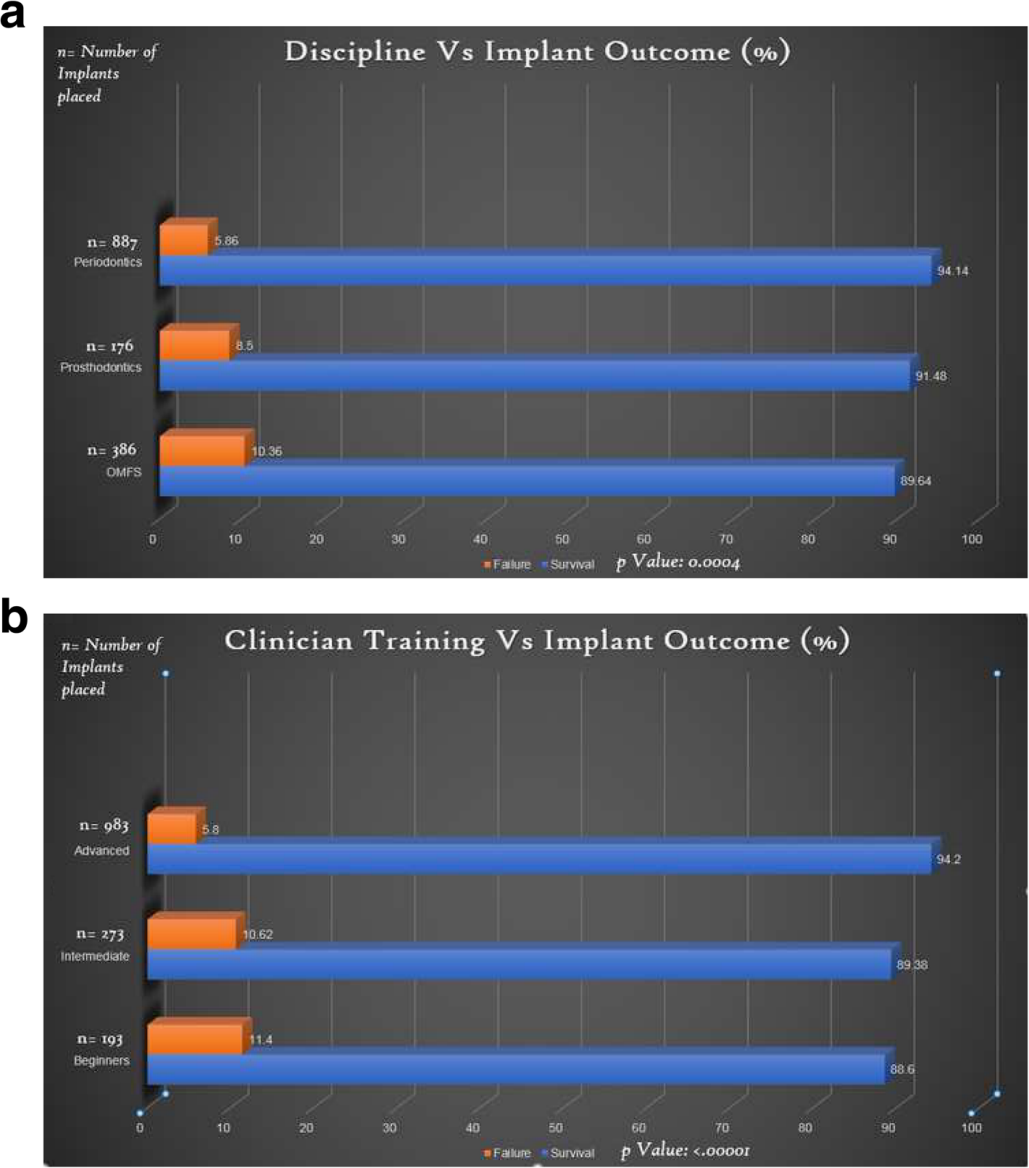
**a** Distribution of dental implant outcome across various disciplines. **b** Distribution of dental implant outcome across various clinician training level

Our analyses indicated that the type of clinician training and level of experience also had a positive association with implant outcome. These results emphasize the importance of appropriate surgical and didactic training obtained over the years in a residency program that ultimately leads to the development of competent surgical skills (Fig. 1). A key limitation of the study was the retrospective timeline of up to 5 years and the fact that the implants included had been present for variable times. This was due to the availability of complete patient records of patients that included follow up for at least 1 year from implant placement. Availability of complete patient records, variation in faculty training, nature of interdisciplinary collaboration and restoration of these implants may have influenced the implant outcome for a particular discipline. Further studies of factors related to types of implant restorations, whether implants were splinted or non-splinted and immediate versus delayed loading are warranted.

## Conclusion

The data analysis results achieved via SAS and MATLAB were identical. We, also, conclude that the level and type of clinician training does have an impact on implant failure rates. However, additional factors need to be considered as they influence the implant survival outcomes. Overall, our findings may help with the standardization of training programs within dental schools where placement of dental implants is part of the curriculum. This could potentially help reduce implant failures and provide more predictable and better implant outcomes for patients treated in dental schools. Further studies will be necessary to identify and analyze the reasons for the differences in implant failure rates so that curriculum changes can be recommended to minimize them.

## Data Availability

The datasets used and/or analyzed during the current study are available from the corresponding author on reasonable request.

## Abbreviations

IRB: Institutional Review Board
LSUHSC: Louisiana State University Health Science Center
OMFS: Oral and Maxillofacial Surgery
